# Obesity and the Impact on Cutaneous Melanoma: Friend or Foe?

**DOI:** 10.3390/cancers12061583

**Published:** 2020-06-15

**Authors:** Lorey K. Smith, Shaghayegh Arabi, Emily J. Lelliott, Grant A. McArthur, Karen E. Sheppard

**Affiliations:** 1 Cancer Research Division, Peter MacCallum Cancer Centre, Melbourne, VIC 3000, Australia; lorey.smith@petermac.org (L.K.S.); Shaghayegh.Arabi@petermac.org (S.A.); emily.lelliott@petermac.org (E.J.L.); grant.mcarthur@petermac.org (G.A.M.); 2 Sir Peter MacCallum Department of Oncology, University of Melbourne, Parkville, VIC 3010, Australia; 3 Department of Biochemistry and Molecular Biology, University of Melbourne, Parkville, VIC 3010, Australia

**Keywords:** melanoma, obesity, targeted therapy, immunotherapy

## Abstract

Excess body weight has been identified as a risk factor for many types of cancers, and for the majority of cancers, it is associated with poor outcomes. In contrast, there are cancers in which obesity is associated with favorable outcomes and this has been termed the “obesity paradox”. In melanoma, the connection between obesity and the increased incidence is not as strong as for other cancer types with some but not all studies showing an association. However, several recent studies have indicated that increased body mass index (BMI) improves survival outcomes in targeted and immune therapy treated melanoma patients. The mechanisms underlying how obesity leads to changes in therapeutic outcomes are not completely understood. This review discusses the current evidence implicating obesity in melanoma progression and patient response to targeted and immunotherapy, and discusses potential mechanisms underpinning these associations.

## 1. Introduction

Cutaneous melanoma is a cancer of melanocytes, the pigment producing cells of the skin. The global incidence of cutaneous melanoma is consistently rising, and Australia has one of the highest rates of melanoma incidences worldwide, with an estimated 15,000 new cases in 2019 (Cancer in Australia 2019: Australian Institute of Health and Welfare). Genetic, host and environmental risk factors have been associated with melanoma incidence. The major environmental risk factor for cutaneous melanoma, is ultraviolet (UV) radiation especially with intermittent patterns of sun exposure [1,2]. Increased number of melanocytic nevi (moles) and fairness of skin are important host risk factors [3,4], and although there is not a strong genetic link, polymorphisms of the melanocortin 1 receptor gene, that are responsible for the different human skin-color phenotypes leads to heightened sensitivity to UV and thus increased susceptibility to cutaneous melanoma [5]. Excess body weight has been identified as a risk factor for many types of cancers [6,7], however the association with cutaneous melanoma incidence is not as strong [7,8,9,10,11]. The discrepancies between these studies may stem from several confounding factors including gender [8,10,12,13], menopausal state [14] and UV exposure [15,16], a clear major driver of cutaneous melanoma [17]. In contrast, several recent studies have indicated that increased body fat is associated with improved survival outcomes in targeted- and immune- therapy treated melanoma patients [13,18,19,20,21]. This review will address the impact of body fat on cutaneous melanoma and current systemic treatments with a focus on cell signalling, metabolism and the immune microenvironment.

## 2. Impact of Body Fat on Melanoma Growth, Metastasis and Cell Signaling Pathways 

**Figure 1:**
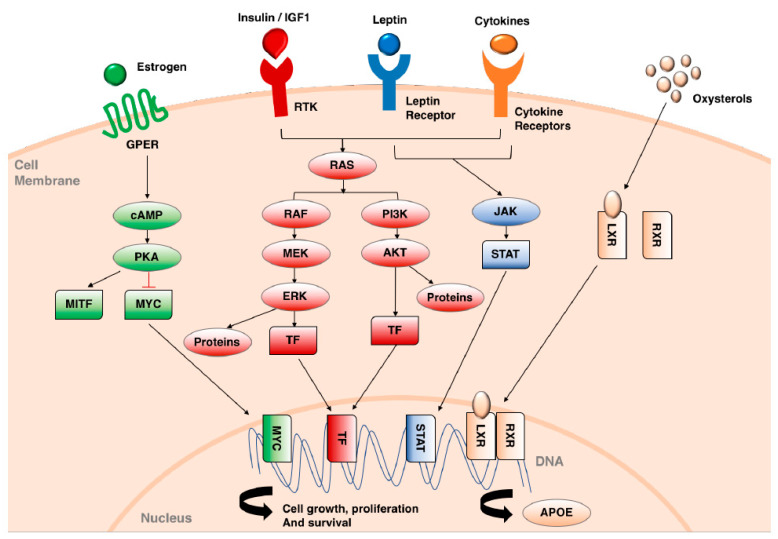
Signaling pathways activated by obesity-induced factors. Circulating factors increased in obesity can activate several signaling pathways that impact on cell growth, proliferation and survival through the regulation of both transcriptional factors and cellular proteins. RTK: receptor tyrosine kinase. GPER: G-protein coupled estrogen receptor. TF: Transcription factor. cAMP: Cyclic adenosine 3′,5′-monophosphate. PKA: protein kinase A. MITF: Microphthalmia-associated transcription factor. MYC: Myc Proto-Oncogene. RAF: RAF family of serine/threonine kinases. MEK: Mitogen-activated protein kinase kinase (also known as MAP2K). ERK: extracellular-signal-regulated kinase (also known as MAPK). PI3K: Phosphoinositide-3-kinase. AKT: Protein kinase B. JAK: Janus kinase. STAT: signal transducer and activator of transcription. LXR: The liver X receptor. RXR: retinoid X receptor. APOE: apolipoprotein E.

The exact molecular mechanisms associated with obesity that lead to cancer development or changes in patient outcomes are not completely understood [22]. Obesity is associated with significant metabolic and endocrine abnormalities, including increased blood levels of growth factors such as insulin, insulin-like growth factor-1 (IGF-1) and leptin that can promote tumor cell growth and survival (Figure 1). Furthermore, there is a growing body of evidence that adipocytes (fat cells) via directly influencing cancer cell metabolism promote tumor growth [23] and cytokines released due to obesity induced chronic low-level inflammation also promotes cancer cell proliferation and survival [24].

In melanoma, obesity is associated with worse outcomes after resection [11] and increased Breslow thickness, which is a prognostic factor in primary melanoma [25], indicating that obesity may be associated with disease aggressiveness. In preclinical melanoma mouse models diet induced obesity increases tumor growth and metastatic spread [26,27,28,29] supporting a role of obesity in promoting melanoma progression. The mechanisms behind the obesity induced increase in melanoma aggressiveness are not completely clear and are probably multifactorial. Adipocytes are lipid-rich, highly secretory cells that release lipids, chemokines, inflammatory factors and adipokines including leptin [30], all of which could impact on melanoma progression. Clinical data suggest that leptin has a role in both melanoma growth [31] and metastasis [32] and this is supported in preclinical studies in which leptin was found to increase melanoma cell proliferation and tumor growth [33,34,35]. Many growth factor receptors are expressed on melanoma cells including receptors for insulin and IGF-1, two key growth factors increased in obese patients. Expression of these receptors correlate with melanoma progression [36] and in preclinical melanoma models they can promote melanoma cell proliferation, survival and migration [37,38,39]. Thus, both preclinical and the limited clinical studies implicate obesity associated growth factors as having the potential to promote melanoma growth and metastasis.

## 3. Impact of Body Fat on Melanoma Metabolism

Cell intrinsic alterations in metabolism are a hallmark of many cancers, including melanoma. While initial studies focused on changes in aerobic glycolysis first identified by Otto Warburg, a growing body of evidence now supports critical roles for other metabolic pathways, including fatty acid synthesis and oxidation [40]. Fatty acids form the structural foundation of cellular and organelle membranes, thus proliferating cells require a large supply of fatty acids to form these structures. The rate limiting step of fatty acid synthesis is catalyzed by fatty acid synthase (FASN), and upregulation of FASN can allow adequate production of the phospholipids to meet the requirements of proliferating cells [41]. FASN expression correlates with poor prognosis in cutaneous melanoma [42], and inhibition of FASN reduces proliferation of B16–F10 melanoma cells in vitro [43] and reduces incidence of metastasis in vivo [44]. In normal tissue, FASN is positively regulated by insulin and hormones such as estrogen and progesterone via induction of the sterol regulatory element-binding protein 1c (SREBPC1c) transcription factor, and is negatively regulated by leptin in response to free fatty acids [41,45,46]. In the context of melanoma, and indeed many other tumor types, SREBPC1c, and consequently FASN expression, is constitutively driven by the mitogen-activated protein kinase (MAPK) and phosphoinositide-3-kinase (PI3K) signaling pathways [41,45]. How increased levels of body fat and obesity, that are frequently associated with alterations in insulin and leptin dependent signaling, influence these signaling networks in the context of melanoma progression is unknown. Further to FASN, multiple genes associated with fatty acid metabolism including lipogenesis and fatty acid oxidation are upregulated in melanomas relative to benign nevi [47]. One of the most significantly upregulated genes in this dataset was carnitine palmitoyltransferase 2 (CPT2) the enzyme critical for translocation of long-chain fatty acids in preparation for β-oxidation. Whilst suggestive of an important role for fatty acid oxidation (FAO) in melanoma progression, the mechanisms underpinning this relationship remain unclear, and the effect of body fat on this association is untested. However, it is predicted that increased fatty acid supply and oxidation can boost cellular ATP under nutrient poor conditions, such as those experienced by metastasizing cancer cells. Together these studies hint at potential cell intrinsic mechanisms that may underpin the relationship between melanoma aggressiveness and obesity and warrant further investigation.

**Figure 2:**
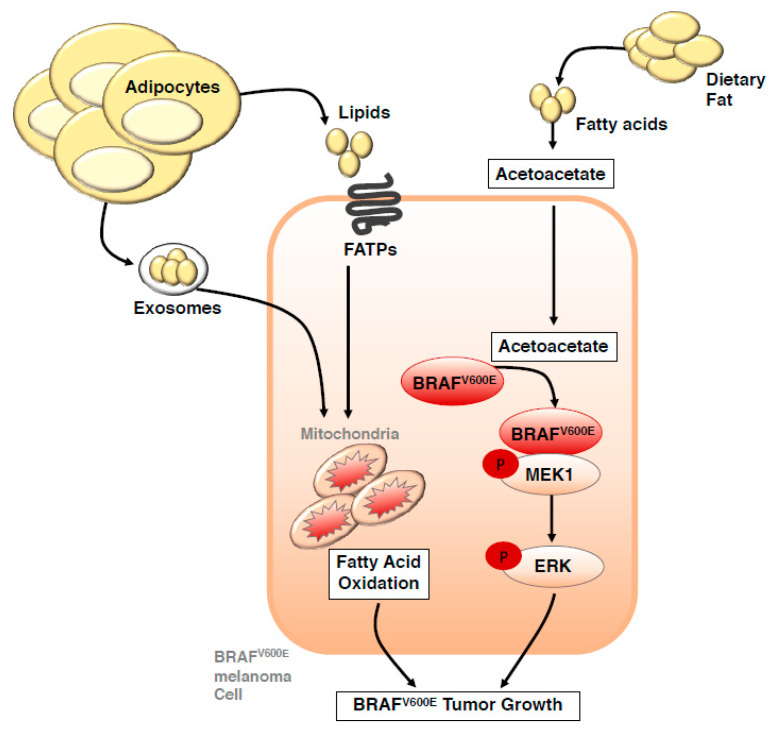
Crosstalk between adipocytes and dietary fat with signalling and metabolism in melanoma cells. Dietary fat and adipocyte-derived lipids have been uncovered as a potential fuel source that can drive melanoma progression. Adipocytes directly transfer lipids to melanoma cells via the fatty acid transporter (FATP; also known as solute carrier family 27, SLC27A) family of proteins and via adipocyte-derived exosomes to promote fatty acid oxidation. Circulating lipids associated with a high-fat diet lead to increased serum levels of acetoacetate, a metabolite that selectively promotes interaction between the mutant BRAF^V600^ protein and MEK1, thereby promoting oncogenic signaling and tumor growth. BRAF: BRAF serine/threonine kinase. MEK: Mitogen activated protein kinase kinase (also known as MAP2K).

More recently cell extrinsic factors such as adipocyte-derived lipids have been uncovered as a potential fuel source that can also drive melanoma progression [48,49]. Advanced, metastatic melanomas frequently grow in subcutaneous tissues that are largely composed of adipocytes. Kwan and colleagues [48] initially found that co-culture of melanoma cells with adipocytes isolated from inguinal adipose tissue in mice facilitated palmitic acid uptake by melanoma cells that stimulated an AKT-dependent increase in melanoma cell proliferation, thus linking cell extrinsic adipocyte derived lipids and cancer cell intrinsic signaling. These observations have recently been extended to in vivo models, whereby stromal adipocytes can directly transfer lipids to melanoma cells via the fatty acid transporter protein (FATP; also known as solute carrier family 27, SLC27A) family (Figure 2) that are over expressed in subsets of melanoma patients [49]. This process can actively promote melanoma invasion and growth, as evidenced by reduced melanoma lipid uptake, invasion and growth following inhibition of FATPs with the small-molecule inhibitor Lipofermata. Further linking lipid uptake from adipocytes with oncogenic signaling in melanoma cells, melanocyte-specific FATP1 expression can cooperate with expression of the BRAF^V600E^ oncogene to accelerate melanoma development in transgenic zebrafish and mouse xenograft studies [49]. Interestingly, a high-fat ketogenic diet can also interact with the BRAF^V600E^ oncogene to promote tumor growth in human melanoma cells implanted into mice [50]. Mechanistically, increased serum levels of acetoacetate are observed in mice on a high fat ketogenic diet, and this metabolite can selectively enhance BRAF^V600E^ mutant-dependent mitogen activated protein kinase kinase (MAP2K; MEK1) activation [51] (Figure 2). This study provides compelling evidence of how cell extrinsic factors such as a high fat diet can directly influence cell-intrinsic signaling pathways, however, how this signaling axis operates in the context of excess body fat still requires investigation. Clinical evidence of a role for lipid uptake in melanoma progression comes from analysis of the Cancer Genome Atlas (TCGA) melanoma patients, whereby Nath and Chan identified a gene signature that included fatty acid uptake genes caveolin-1 (CAV1) and cluster of differentiation 36 (CD36), and the fatty acid oxidation gene carnitine palmitoyltransferase 1C (CPT1C), that predicts significantly worse overall survival in melanomas enriched for this signature [52]. Supporting this clinical observation, depletion of CD36 reduces propensity of melanoma cells to metastasize in immune compromised mice [53]. Notably, at least in the context of oral carcinoma, palmitic acid or a high-fat diet specifically boosts metastatic potential of CD36^+^ metastasis-initiating cells in a CD36-dependent manner, suggesting that metastasis-initiating cells may rely on dietary lipids to promote metastasis. However, this specific association between dietary fat, CD36 and metastasis remains to be tested in melanoma.

Cross talk between adipocytes and melanoma cells can also occur across more distant proximities via adipocyte-derived exosomes that carry proteins and substrates utilised as fuel that drive fatty acid oxidation, and thereby influence melanoma cell metabolism [54] (Figure 2). Intriguingly, in the context of obesity, adipocytes secrete more extracellular vesicles (EV), and exposure of melanoma cells to these EVs further potentiates effects on melanoma migration when compared to EVs isolated from non-obese counterparts. Mechanistically, the heightened effect of EVs in the context of obesity appears dependent on increased access to fatty acid fuels, rather than differences in FAO related proteins [55]. These studies clearly demonstrate additional ways that adipocytes within the tumor microenvironment, and also beyond, can influence melanoma cell intrinsic metabolism, and might in part explain why some obese melanoma patients experience a more aggressive disease than their non-obese counterparts.

## 4. Impact of Body Fat on the Melanoma Immune Microenvironment 

Cutaneous melanoma is the most highly mutated malignancy [56] resulting in the generation of novel epitopes which contribute to its immunogenicity and the success of immunotherapy for its treatment [57,58,59,60]. However, predicted neoantigen load does not correlate with T cell infiltration in melanoma [61] and is not a robust predictor of immunotherapy efficacy [62], suggesting that additional factors determine T cell responses. Melanomas that progress have likely escaped immune control. Tumor immune escape is the final phase in the immunoediting process, with elimination and equilibrium being the preceding phases [63]. During these phases, tumor immunogenicity is edited, and immunosuppressive mechanisms enable disease progression. The elimination phase involves tumor cell recognition and killing and engages both innate and adaptive immune cells. Following this an equilibrium phase is reached in which tumor cells that have reduced immunogenicity and that can evade both immune recognition and killing are selected for [64]. This selection process may involve the emergence of non-immunogenic rare clones that were always present in the tumor or clones that evolved mechanisms to evade immune surveillance. Such mechanisms include loss of antigen presentation or co-stimulatory molecules, expression of immune inhibitory molecules (Programmed death-ligand 1; PD-L1) or secretion of cytokines such as macrophage colony-stimulating factor (M-CSF), transforming growth factor β (TGF-β) and interleukin 10 (IL-10) [65,66,67,68,69,70,71]. Tumor cells which have acquired resistance to immune mediated elimination then enter the escape phase, where tumor cell proliferation outweighs immune control leading to disease progression.

Obesity induces a chronic systemic inflammation state [72] leading to changes in immune cell populations and impaired anti-tumor immune responses. Chronic inflammation compromise tumor immunosurveillance by significantly attenuating the number and activity of natural killer (NK) and NK-T cells [73], and promotes further tumor progression by impairing dendritic cell (DC) function and subsequent activation of T cell responses [74]. T cell number and diversity are also restricted in obesity due to thymic aging [75,76] and T cell dysfunction is prominent as a result of the chronic inflammation [21]. Obesity can also increase tumor infiltrating immune suppressor cells including macrophages and myeloid derived suppressor cells (MDSCs) [77]. Moreover, adipose tissue generates pro-inflammatory factors, that impact on tumor cell proliferation and survival [78]. Obesity not only induces an increase in tumor infiltration of MDSCs but also MDSC expression of PD-L1 thereby inhibiting anti-tumor responses and promoting tumor growth and metastasis [77]. In a preclinical melanoma model diet induced obesity led to a leptin-dependent decrease in functional DCs and tumor infiltrating CD8+ T cells indicating impairment of an anti-tumor response [79]. A recent study in a mouse melanoma model demonstrated that the frequency of CD8+ T cells expressing exhaustion markers (PD-1, TIM3 and LAG3) were significantly higher both at the tumor site and systemically in obese mice compared to their lean counterparts implicating leptin as the mediator [21]. Further supporting a role of obesity-induced T cell exhaustion in melanoma is the increase in gene expression of inhibitory molecules PD-1, TIGIT, EOMES, TIM3 and LAG3 found in melanoma patients with a high body mass index (BMI) [21]. Moreover, immunosuppressive cytokines and angiogenic factors such as vascular endothelial growth factor (VEGF) produced by melanoma cells, infiltrated macrophages, and adipose-derived stem cells have all been shown to promote melanoma growth [27,80,81].

With respect to T cell intrinsic metabolism, T cell activation induces dramatic physiological changes associated with exponential proliferation required at the height of an immune response. To meet the energetic requirements of these new tasks T cells undergo metabolic reprograming in order to generate sufficient biomass and ATP [82]. T cells exhibit a plethora of metabolic phenotypes depending on subclass and activation state. Activated CD4+ and CD8+ effector T cells upregulate glucose transporters and glycolytic enzymes to adopt a glycolytic phenotype, whilst CD8+ memory T cells catabolize fatty acids and utilize OXPHOS to produce ATP [82]. Interestingly, modulation of fatty acid metabolism has been shown to enhance CD8+ memory T cell function [83], however paradoxically, the fatty acid oxidation gene *CPT1A* has also been linked with T cell dysfunction, whereby CPT1A is upregulated in early stage exhausted T cells following viral infection [84]. Interestingly, in this model of chronic lymphocytic choriomeningitis virus (LCMV) infection, alterations in CD8+ T cell metabolism occurred downstream of PD-1 and involved downregulation of the master regulator of mitochondrial metabolism, PGC1A. Demonstrating causality, ectopic expression of PGC1A was sufficient to partially reverse the observed metabolic alterations and enhanced function of exhausted CD8+ T cells, whilst T cell reinvigoration via anti-PDL1 reprogrammed metabolism in subsets of the exhausted T cells [84]. In the context of diet-induced obese mice bearing melanoma, CPT1A is also upregulated in CD8+ T cells in association with other biomarkers of T cell exhaustion, and this appears to be mediated via leptin signaling as administration of leptin to leptin-deficient mice was sufficient to induce CPT1A expression in CD8+ T cell populations [21]. Although the direct role of CPT1A mediated fatty acid oxidation in T cell exhaustion in the context of melanoma has not been specifically investigated, it is tempting to speculate that this axis may be exacerbated in obese versus non-obese melanoma patients and may contribute to melanoma progression due to increased levels of T cell exhaustion. Conversely, modulation of this axis may also represent an opportunity to enhance response to immunotherapies that exploit the PD-1/PDL-1 immune checkpoint.

There is a growing body of evidence that suggests the innate immune system has a memory component that allows these cells to respond more robustly to future challenges. This “innate immune memory” or “trained immunity” is mediated by epigenetic and metabolic reprogramming of innate immune cells [85,86], and in contrast to adaptive immunity, the second challenge does not have to be the same as the first. In a recent study in mice, a Western-like diet induced systemic inflammation and reprogramed bone marrow myeloid progenitors resulting in trained immunity [87]. The heightened inflammatory responses to subsequent innate immune stimuli persisted after the mice had been switched to a standard chow diet. Similarly, in obesity the persistent activation of adipose tissue macrophages is maintained even after weight loss [88]. Thus, the chronic low-level inflammation associated with obesity is likely in part a function of trained immunity. How obesity induced trained immunity impacts on tumor cells is unknown. However, in melanoma [89], as well as other cancers [90,91] vaccination with Bacillus Calmette-Guerin (BCG) can lead to anti-tumor responses which are likely due to its capacity to induce trained immunity [92], indicating that in some cases trained immunity may be beneficial. 

## 5. The Impact of Body Fat on Melanoma Treatment

Immune checkpoint inhibitors and molecular targeted therapies directed against BRAF and MEK, two kinases in the mitogen activated protein kinase pathway (MAPK), have revolutionised melanoma treatment. With the discovery of the prevalence of a specific activating mutation in BRAF (V600) [17] selective mutant BRAF inhibitors were developed. In parallel, immune checkpoint inhibitors that target two key immune inhibitory molecules, cytotoxic T-lymphocyte antigen 4 (CTLA4) and PD-1 were also developed. As described above obesity can impact tumor signaling pathways and the tumor immune microenvironment, in this section the impact of adipose tissue on these therapies will be discussed (Table 1).

### 5.1. Targeted Therapy

Currently, the standard-of-care for BRAF-mutant melanoma is an upfront combination of a BRAF and MEK inhibitor (BRAF/MEKi), of which there are three combinations approved by the Food and Drug Administration (FDA): dabrafenib/trametinib, vemurafenib/cobimetinib and encorafenib/binimetinib. This combinational therapy is highly tolerated and the initial response rates are remarkable with 90% of patients having some degree of response [93] that is associated with rapid improvements in symptoms and improved survival [94,95,96]. However, resistance to this therapy often develops leading to relapse and making long term disease control a challenge for most patients. The 5-year overall survival rates for the dabrafenib plus trametinib combination in BRAF mutant melanoma patients is 34% [94].

The mutation in BRAF locks the kinase into an active conformation leading to constitutive activation of the MAPK pathway and as consequence increased cell growth, proliferation and survival (Figure 1). Resistance to BRAF and BRAF/MEK targeted therapies is thought to occur in two phases. The first phase is a drug tolerant state which involves reversible transcriptional and translational reprogramming leading to invasive mesenchymal phenotypic switch and metabolic changes [97,98], followed by non-reversible drug resistance leading to tumor outgrowth. Multiple mechanisms result in resistance [99] including reactivation of extracellular signal regulated kinase (ERK)-signaling, activation of the PI3K pathway and activation of a receptor tyrosine kinases (RTKs).

A recent study in metastatic melanoma patients demonstrated that obesity is associated with significantly improved responses to both targeted and immunotherapy in male but not female patients. At the 2-year mark, male obese patients on targeted dabrafenib and trametinib therapy had an overall survival of 64% compared to 35% in normal weight male patients with overweight patients falling in between. Furthermore, progression free survival was also improved, 15.7 months for obese individuals versus 9.6 months for normal [13]. How obesity increases the efficacy of MAPK pathway targeted therapy in melanoma patients is unknown. Obesity leads to metabolic disturbances resulting in increased blood levels of insulin and IGF-1, a meta-inflammation state associated with elevated levels of circulating cytokines and adipocytes produce adipokines such as leptin and estrogen [100]. Many of these factors activate pathways (Figure 1) that are associate with resistance to targeted therapy thus the obesity associated increase in BRAF/MEK targeted therapy efficacy is counterintuitive. Two receptors that may be activated in obesity and could explain the increased efficacy of targeted therapy in obese patients are liver X receptor (LXR) and G protein-coupled estrogen receptor (GPER). LXRs bind oxysterols, bioactive lipids derived from cholesterol, which are elevated in obesity [101,102]. Activation of LXR and the subsequent induction of tumoral and stromal apolipoprotein-E (ApoE) decreases melanoma proliferation and metastasis and can increase the efficacy of vemurafenib, a BRAF inhibitor [103,104]. Furthermore, LXR activation can directly decrease cell proliferation by increasing levels of the cell cycle inhibitor p27 and by inducing cholesterol efflux, which disrupts lipid rafts and thus receptor tyrosine kinase (RTK) [105] and AKT signalling [106], both of which are implicated in resistance to BRAF/MEK targeted therapy. GPERs are expressed on melanoma cells whereas classical estrogen receptors are not [107]. Estrogen the activator of GPERs is elevated in obese male patients and postmenopausal women due to adipocyte aromatase activity [108]. In melanoma activation of GPERs leads to depletion of the transcription factor c-MYC [109] an oncogene that has been shown to be a convergence point for pathways conferring resistance to BRAF/MEK inhibitors [110]. Thus activation of either LXRs and/or GPERs may counteract the emergence of resistance to BRAF/MEK targeted therapy delaying tumor progression and prolonging overall survival. 

Treatment of BRAF^V600E^ melanoma cells with BRAF and MEK targeted therapies extensively reprograms cell intrinsic metabolism and this influences clinical response. During the initial phase of treatment, a dramatic decrease in glucose utilisation and glycolytic flux is observed [111], and this correlates with clinical response in patients. Subsequently, an adaptive upregulation of oxidative mitochondrial metabolism occurs via the master mitochondrial transcription factor PGC1α that limits response to BRAF inhibition [112]. Induction of oxidative phosphorylation and mitochondrial biogenesis biomarkers has been identified in 30–50% of BRAF^V600E^ melanomas with both de novo and acquired resistance to MAPK pathway inhibitors [113], and inhibition of mitochondrial biogenesis eradicated intrinsically resistant cells and improved efficacy of MAPK pathway inhibitors [114]. These data clearly demonstrate melanoma cell intrinsic metabolism influences response to targeted therapies, however, how changes in systemic metabolism and increased adiposity associated with obesity influences this relationship remains almost completely unexplored. One study has described a negative effect of adipocytes on targeted therapy response in melanoma cells in vitro [115], and this effect was reversed upon inhibition of leptin signaling, however this observation in cell lines is contrary to the positive obesity/targeted therapy interaction observed in patients. Moreover, preclinical analysis of adipocyte crosstalk with BRAF^V600E^ signaling via the FATP/ SLC27A fatty acid transporters would be predicted to abrogate response of obese patients to MAPK pathway targeted therapies based on potentiation of BRAF^V600E^ driven melanoma progression by adipocyte-melanoma cell crosstalk [49]. Similarly, increased dietary fat has a pathogenic role in BRAF^V600E^-expressing melanoma [50] which would be predicted to blunt MAPK pathway targeted therapies.

Beyond cell intrinsic metabolism, alterations in systemic metabolism can influence patient responses to targeted therapies. In normal physiological settings, the PI3K pathway functions as a nutrient sensor and is activated by binding of insulin and IGF to cell-surface receptors. Systemic regulation of this pathway is altered in the setting of obesity, frequently manifesting in insulin resistance. This pathway is also frequently activated in many types of cancer, and PI3K pathway inhibitors are under investigation as a cancer treatment. Interestingly, efficacy of PI3K inhibitors appears to be limited due to systemic glucose-insulin feedback caused by targeted inhibition of the PI3K pathway, that is sufficient to reactivate PI3K signalling in tumor cells across multiple tumor models in mice [116]. This insulin feedback can be prevented in the context of a high fat ketogenic diet to greatly enhance the efficacy of PI3K inhibition. Given PI3K signaling is associated with resistance to MAPK pathway targeted therapies in melanoma [117], how systemic metabolic signaling via PI3K in the context of obesity influences MAPK targeted therapy response warrants further investigation. Another potential point of intersection between these signaling pathways and cell intrinsic and systemic metabolism, is regulation of the SREBPC1c transcription factor that controls lipid metabolism via targets such as FASN (discussed above). Under normal physiological conditions the SREBPC1c-FASN axis is positively regulated by insulin and negatively regulated by leptin in response to free fatty acids [41,45,46]. Leptin has been linked with the positive obesity/targeted therapy association in melanoma patients, but whether the SREBPC1c-FASN axis plays a role in this association is unknown. Notably, SREBPC1c is also subject to regulation by progesterone and estrogen thus systemic regulation of SREBPC1c mediated metabolism may potentially contribute to observed sex-based differences in the positive effects of obesity on targeted therapy in melanoma patients.

Another critical aspect of targeted therapy response in melanoma patients is the upregulation of antigen expression and increased infiltration of T cells which is associated with tumor shrinkage [118,119]. Interestingly, immune cell metabolism is directly linked with differentiation status and function in the context of anti-tumor immunity [120,121], and emerging evidence now suggests that lipids released from adipocytes can be used as a fuel for immune cells and promote their differentiation [122]. Flaherty and colleagues recently found that adipocytes release exosome-sized, lipid-filled vesicles that become a source of lipid for local macrophages that drive their differentiation, and notably, obese adipocytes release more of these lipid filled vesicles than non-obese adipocytes [122]. It is therefore plausible to consider that increased release of lipids from adipocytes in obese versus non-obese melanoma patients can impact on targeted therapy induced immune responses by fueling immune cell populations. Interestingly, obese melanoma patients also respond better to immunotherapy (discussed below), however it is more difficult to reconcile this hypothesis with sex-based differences observed in the positive association between obesity and therapy.

### 5.2. Immunotherapy

There are currently seven FDA-approved immunotherapy options for melanoma that fall into two classes, oncolytic virus therapy and immune modulators, which includes the immune checkpoint inhibitors that block key immunological pathways that mediate tumor escape. Of these therapies the immune checkpoint inhibitors have shown excellent efficacy in melanoma with overall survival of 52% at 5-years when treated with a combination of ipilimumab, that targets CTLA-4 and nivolumab that targets PD-1 [60]. CTLA-4 and PD-1 checkpoint inhibitors work via distinct mechanisms with a common outcome of enhancing anti-tumor T cell immunity. Anti-CTLA-4 de-represses antigen-cross presentation interactions between T cells and dendritic cells at the priming phase of T cell activation, while anti-PD-1 blocks inhibitory interactions between activated T cells and the tumor cells they target [124]. However, severe toxicity is a major issue for approximately 60% of patients treated with combined immunotherapy [125]. In melanoma there have been several recent clinical studies demonstrating a favorable association between a high body mass index and immune check point therapy [13,18,19,21,123]. Ipilimumab (specific monoclonal antibody against CTLA-4) with [13] or without chemotherapy [123] led to significantly enhanced progression-free survival (PFS) and overall survival (OS), in overweight melanoma patients (BMI ≥ 25) when compared to normal-weight patients. This association was also observed with either PD1 or PD-L1 check point inhibitor therapy [13,18,19], with two studies indicating that significant benefit is only in overweight male melanoma patients [13,18]. 

The role of obesity in immune checkpoint therapy efficacy is not clear. Anti-PD-1 therapy is more efficacious in melanoma-bearing obese mice compared to their normal-weight counterparts [21]. The improved response was associated with increased number and function of tumor infiltrating CD8+ T cells together with a decrease in PD-1 expressing T cells; indicating that anti-PD1 therapy overcame obesity-driven T-cell exhaustion. Increased leptin in the obese mice was implicated in mediating the PD-1 expression and T cell exhausted phenotype [21]. In contrast, treatment response to CTLA-4 blockade was not improved in diet-induced obese mice, however when leptin was neutralized in this model an increase in the co-stimulatory molecule CD86 was observed as was the response to anti-CTLA therapy [79]. In both models, leptin is implicated in inducing an immunosuppressive microenvironment by increasing PD-1 expression and decreasing immune-stimulatory molecules. The leptin induced increase in PD-1 expression on T cells allows for increased efficacy to anti-PD1 therapy but the down regulation of co-stimulatory molecules results in decreased anti-CTLA efficacy. However, the effects of leptin on the immune tumor microenvironment is likely to be more complex with its reported multiple effects on the regulation of both innate and adaptive immune responses [126]. Furthermore, obesity also impacts on gut microbiome diversity [127] which can play a role in response to immune checkpoint blockade efficacy in cancer including melanoma [128,129].

The more favourable prognosis of female melanoma patients compared to males [10,130,131] and the difference in response to immunotherapy observed between obese male and female melanoma patients implicates sex hormone status as a factor in outcome. The obvious candidate is estrogen that is produced by adipocytes, which is the main source of estrogen in males. Estrogen can also have direct effects on both innate and adaptive immunity [132] and these may promote immunotherapy outcomes. As mentioned earlier classical estrogen receptors are not expressed on melanoma but GPERs are and when activated can drive melanoma differentiation and decrease MYC driven PD-L1 expression potentially increasing tumor susceptibility to T cell control [107]. In mouse melanoma models activation of GPERs also synergizes with anti-PD1 therapy inducing tumor regression, infiltrating T cells, HLA expression and immune memory [109]. 

## 6. Conclusions

Excess body fat is a complex metabolic disorder that can potentially impact on melanoma growth and immune surveillance at many levels. Why obese melanoma patients respond better to both targeted- and immune-therapy remains unclear. Many factors increased in obesity would promote melanoma growth and similar to the concept of “oncogenic addiction” this could make them more susceptible to targeted therapy. Similarly, the immune-suppressive microenvironment induced by obesity could confer greater sensitivity to immune checkpoint inhibitors. Many additional complex cellular processes induced in obesity may also impact on melanoma, including gender and the gut microbiome. 

## Figures and Tables

**Table 1:** The association of high body mass index with melanoma treatment.

Reference	Cohort BMI kg/m^2^	Immunotherapy	Targeted Therapy	Chemotherapy
[13]	Normal: <25 Obese: >30	Improved PFS and OS in obese melanoma cohort. Improved PFS and OS in male obese patients. No association in female obese patients. (ipilimumab, pembrolizumab, nivolumab, or atezolizumab)	Improved PFS and OS in obese melanoma cohort. Improved PFS and OS in male obese patients. No association in female obese patients. (dabrafenib + trametinib and vemurafenib + cobimetinib)	No association between BMI and clinical outcomes
[19]	Normal: 18.5–24.9 Overweight/obese: >25	Improved PFS and OS in obese melanoma cohort (anti-PD-1/PD-L1 inhibitors)	N/A	N/A
[123]	Normal: 18.5–25 Overweight/obese: 25–35	Significantly higher response rate and a trend for longer OS in overweight/obese melanoma cohort (Ipilimumab)	N/A	N/A
[21]	Normal/overweight: ˂30 Obese: ≥30	Improved PFS and OS in a cohort of obese advanced cancer patients including melanoma (anti-PD-1/PD-L1 inhibitors)	N/A	N/A
[18]	Normal: 18.5–25 Overweight/obese: 25–35	Improved PFS and OS in overweight/obese melanoma cohort. Association was predominantly driven by males (pembrolizumab, nivolumab, or nivolumab plus ipilimumab)	N/A	N/A

Abbreviations: BMI, body mass index. PFS, Progression free survival. OS, Overall survival. PD-1, Programmed cell death protein 1. PD-L1, Programmed death-ligand 1. N/A, not applicable.

## References

[1] Armstrong B.K., Kricker A. (1993). How much melanoma is caused by sun exposure?. Melanoma Res..

[2] Elwood J.M., Gallagher R.P. (1998). Body site distribution of cutaneous malignant melanoma in relationship to patterns of sun exposure. Int. J. Cancer.

[3] Bauer J., Garbe C. (2003). Acquired melanocytic nevi as risk factor for melanoma development. A comprehensive review of epidemiological data. Pigment Cell Res..

[4] Rastrelli M., Tropea S., Rossi C.R., Alaibac M. (2014). Melanoma: Epidemiology, risk factors, pathogenesis, diagnosis and classification. Vivo.

[5] Sturm R.A. (2002). Skin colour and skin cancer—MC1R, the genetic link. Melanoma Res..

[6] Renehan A.G., Roberts D.L., Dive C. (2008). Obesity and cancer: Pathophysiological and biological mechanisms. Arch. Physiol. Biochem..

[7] Lauby-Secretan B., Scoccianti C., Loomis D., Grosse Y., Bianchini F., Straif K. (2016). International Agency for Research on Cancer Handbook Working G. Body Fatness and Cancer--Viewpoint of the IARC Working Group. N. Engl. J. Med..

[8] Dobbins M., Decorby K., Choi B.C. (2013). The Association between Obesity and Cancer Risk: A Meta-Analysis of Observational Studies from 1985 to 2011. ISRN Prev. Med..

[9] Tang J.Y., Henderson M.T., Hernandez-Boussard T., Kubo J., Desai M., Sims S.T., Aroda V., Thomas F., McTiernan A., Stefanick M.L. (2013). Lower skin cancer risk in women with higher body mass index: The women’s health initiative observational study. Cancer Epidemiol. Prev. Biomark..

[10] Sergentanis T.N., Antoniadis A.G., Gogas H.J., Antonopoulos C.N., Adami H.O., Ekbom A., Petridou E.T. (2013). Obesity and risk of malignant melanoma: A meta-analysis of cohort and case-control studies. Eur. J. Cancer.

[11] Fang S., Wang Y., Dang Y., Gagel A., Ross M.I., Gershenwald J.E., Cormier J.N., Wargo J., Haydu L.E., Davies M.A. (2017). Association between Body Mass Index, C-Reactive Protein Levels, and Melanoma Patient Outcomes. J. Invest. Derm..

[12] Olsen C.M., Green A.C., Zens M.S., Stukel T.A., Bataille V., Berwick M., Elwood J.M., Gallagher R., Holly E.A., Kirkpatrick C. (2008). Anthropometric factors and risk of melanoma in women: A pooled analysis. Int. J. Cancer.

[13] McQuade J.L., Daniel C.R., Hess K.R., Mak C., Wang D.Y., Rai R.R., Park J.J., Haydu L.E., Spencer C., Wongchenko M. (2018). Association of body-mass index and outcomes in patients with metastatic melanoma treated with targeted therapy, immunotherapy, or chemotherapy: A retrospective, multicohort analysis. Lancet Oncol..

[14] Reeves G.K., Pirie K., Beral V., Green J., Spencer E., Bull D., Million Women Study C. (2007). Cancer incidence and mortality in relation to body mass index in the Million Women Study: Cohort study. BMJ.

[15] Gallus S., Naldi L., Martin L., Martinelli M., La Vecchia C. (2006). Oncology Study Group of the Italian Group for Epidemiologic Research in D. Anthropometric measures and risk of cutaneous malignant melanoma: A case-control study from Italy. Melanoma Res..

[16] Shors A.R., Solomon C., McTiernan A., White E. (2001). Melanoma risk in relation to height, weight, and exercise (United States). Cancer Causes Control.

[17] Hodis E., Watson I.R., Kryukov G.V., Arold S.T., Imielinski M., Theurillat J.P., Nickerson E., Auclair D., Li L., Place C. (2012). A landscape of driver mutations in melanoma. Cell.

[18] Naik G.S., Waikar S.S., Johnson A.E.W., Buchbinder E.I., Haq R., Hodi F.S., Schoenfeld J.D., Ott P.A. (2019). Complex inter-relationship of body mass index, gender and serum creatinine on survival: Exploring the obesity paradox in melanoma patients treated with checkpoint inhibition. J. Immunother. Cancer.

[19] Cortellini A., Bersanelli M., Buti S., Cannita K., Santini D., Perrone F., Giusti R., Tiseo M., Michiara M., Di Marino P. (2019). A multicenter study of body mass index in cancer patients treated with anti-PD-1/PD-L1 immune checkpoint inhibitors: When overweight becomes favorable. J. Immunother. Cancer.

[20] Donnelly D., Bajaj S., Yu J., Hsu M., Balar A., Pavlick A., Weber J., Osman I., Zhong J. (2019). The complex relationship between body mass index and response to immune checkpoint inhibition in metastatic melanoma patients. J. Immunother. Cancer.

[21] Wang Z., Aguilar E.G., Luna J.I., Dunai C., Khuat L.T., Le C.T., Mirsoian A., Minnar C.M., Stoffel K.M., Sturgill I.R. (2019). Paradoxical effects of obesity on T cell function during tumor progression and PD-1 checkpoint blockade. Nat. Med..

[22] Khandekar M.J., Cohen P., Spiegelman B.M. (2011). Molecular mechanisms of cancer development in obesity. Nat. Rev. Cancer.

[23] Cao Y. (2019). Adipocyte and lipid metabolism in cancer drug resistance. J. Clin. Investig..

[24] Renehan A.G., Zwahlen M., Egger M. (2015). Adiposity and cancer risk: New mechanistic insights from epidemiology. Nat. Rev. Cancer.

[25] Skowron F., Berard F., Balme B., Maucort-Boulch D. (2015). Role of obesity on the thickness of primary cutaneous melanoma. J. Eur. Acad. Dermatol. Venereol..

[26] Erickson K.L. (1984). Dietary fat influences on murine melanoma growth and lymphocyte-mediated cytotoxicity. J. Natl. Cancer Inst..

[27] Jung J.I., Cho H.J., Jung Y.J., Kwon S.H., Her S., Choi S.S., Shin S.H., Lee K.W., Park J.H. (2015). High-fat diet-induced obesity increases lymphangiogenesis and lymph node metastasis in the B16F10 melanoma allograft model: Roles of adipocytes and M2-macrophages. Int. J. Cancer.

[28] Pandey V., Vijayakumar M.V., Ajay A.K., Malvi P., Bhat M.K. (2012). Diet-induced obesity increases melanoma progression: Involvement of Cav-1 and FASN. Int. J. Cancer.

[29] Malvi P., Chaube B., Pandey V., Vijayakumar M.V., Boreddy P.R., Mohammad N., Singh S.V., Bhat M.K. (2015). Obesity induced rapid melanoma progression is reversed by orlistat treatment and dietary intervention: Role of adipokines. Mol. Oncol..

[30] Wu Q., Li B., Li Z., Li J., Sun S., Sun S. (2019). Cancer-associated adipocytes: Key players in breast cancer progression. J. Hematol. Oncol..

[31] Gogas H., Trakatelli M., Dessypris N., Terzidis A., Katsambas A., Chrousos G.P., Petridou E.T. (2008). Melanoma risk in association with serum leptin levels and lifestyle parameters: A case-control study. Ann. Oncol..

[32] Oba J., Wei W., Gershenwald J.E., Johnson M.M., Wyatt C.M., Ellerhorst J.A., Grimm E.A. (2016). Elevated Serum Leptin Levels are Associated with an Increased Risk of Sentinel Lymph Node Metastasis in Cutaneous Melanoma. Medicine.

[33] Ellerhorst J.A., Diwan A.H., Dang S.M., Uffort D.G., Johnson M.K., Cooke C.P., Grimm E.A. (2010). Promotion of melanoma growth by the metabolic hormone leptin. Oncol. Rep..

[34] Amjadi F., Mehdipoor R., Zarkesh-Esfahani H., Javanmard S.H. (2016). Leptin serves as angiogenic/mitogenic factor in melanoma tumor growth. Adv. Biomed. Res..

[35] McMurphy T., Xiao R., Magee D., Slater A., Zabeau L., Tavernier J., Cao L. (2014). The anti-tumor activity of a neutralizing nanobody targeting leptin receptor in a mouse model of melanoma. PLoS ONE.

[36] Kanter-Lewensohn L., Dricu A., Girnita L., Wejde J., Larsson O. (2000). Expression of insulin-like growth factor-1 receptor (IGF-1R) and p27Kip1 in melanocytic tumors: A potential regulatory role of IGF-1 pathway in distribution of p27Kip1 between different cyclins. Growth Factors.

[37] Le Coz V., Zhu C., Devocelle A., Vazquez A., Boucheix C., Azzi S., Gallerne C., Eid P., Lecourt S., Giron-Michel J. (2016). IGF-1 contributes to the expansion of melanoma-initiating cells through an epithelial-mesenchymal transition process. Oncotarget.

[38] Hilmi C., Larribere L., Giuliano S., Bille K., Ortonne J.P., Ballotti R., Bertolotto C. (2008). IGF1 promotes resistance to apoptosis in melanoma cells through an increased expression of BCL2, BCL-X(L), and survivin. J. Investig. Derm..

[39] Assa-Kunik E., Fishman D., Kellman-Pressman S., Tsory S., Elhyany S., Baharir O., Segal S. (2003). Alterations in the expression of MHC class I glycoproteins by B16BL6 melanoma cells modulate insulin receptor-regulated signal transduction and augment [correction of augments] resistance to apoptosis. J. Immunol..

[40] Fischer G.M., Vashisht Gopal Y.N., McQuade J.L., Peng W., DeBerardinis R.J., Davies M.A. (2018). Metabolic strategies of melanoma cells: Mechanisms, interactions with the tumor microenvironment, and therapeutic implications. Pigment Cell Melanoma Res..

[41] Menendez J.A., Lupu R. (2007). Fatty acid synthase and the lipogenic phenotype in cancer pathogenesis. Nat. Rev. Cancer.

[42] Innocenzi D., Alo P.L., Balzani A., Sebastiani V., Silipo V., La Torre G., Ricciardi G., Bosman C., Calvieri S. (2003). Fatty acid synthase expression in melanoma. J. Cutan. Pathol..

[43] Zecchin K.G., Rossato F.A., Raposo H.F., Melo D.R., Alberici L.C., Oliveira H.C., Castilho R.F., Coletta R.D., Vercesi A.E., Graner E. (2011). Inhibition of fatty acid synthase in melanoma cells activates the intrinsic pathway of apoptosis. Lab. Investig..

[44] Carvalho M.A., Zecchin K.G., Seguin F., Bastos D.C., Agostini M., Rangel A.L., Veiga S.S., Raposo H.F., Oliveira H.C., Loda M. (2008). Fatty acid synthase inhibition with Orlistat promotes apoptosis and reduces cell growth and lymph node metastasis in a mouse melanoma model. Int. J. Cancer.

[45] Kersten S. (2001). Mechanisms of nutritional and hormonal regulation of lipogenesis. EMBO Rep..

[46] Kim J.B., Sarraf P., Wright M., Yao K.M., Mueller E., Solanes G., Lowell B.B., Spiegelman B.M. (1998). Nutritional and insulin regulation of fatty acid synthetase and leptin gene expression through ADD1/SREBP1. J. Clin. Investig..

[47] Sumantran V.N., Mishra P., Sudhakar N. (2015). Microarray analysis of differentially expressed genes regulating lipid metabolism during melanoma progression. Indian J. Biochem. Biophys..

[48] Kwan H.Y., Fu X., Liu B., Chao X., Chan C.L., Cao H., Su T., Tse A.K., Fong W.F., Yu Z.L. (2014). Subcutaneous adipocytes promote melanoma cell growth by activating the Akt signaling pathway: Role of palmitic acid. J. Biol. Chem..

[49] Zhang M., Di Martino J.S., Bowman R.L., Campbell N.R., Baksh S.C., Simon-Vermot T., Kim I.S., Haldeman P., Mondal C., Yong-Gonzales V. (2018). Adipocyte-Derived Lipids Mediate Melanoma Progression via FATP Proteins. Cancer Discov..

[50] Xia S., Lin R., Jin L., Zhao L., Kang H.B., Pan Y., Liu S., Qian G., Qian Z., Konstantakou E. (2017). Prevention of Dietary-Fat-Fueled Ketogenesis Attenuates BRAF V600E Tumor Growth. Cell Metab..

[51] Kang H.B., Fan J., Lin R., Elf S., Ji Q., Zhao L., Jin L., Seo J.H., Shan C., Arbiser J.L. (2015). Metabolic Rewiring by Oncogenic BRAF V600E Links Ketogenesis Pathway to BRAF-MEK1 Signaling. Mol. Cell.

[52] Nath A., Chan C. (2016). Genetic alterations in fatty acid transport and metabolism genes are associated with metastatic progression and poor prognosis of human cancers. Sci. Rep..

[53] Pascual G., Avgustinova A., Mejetta S., Martin M., Castellanos A., Attolini C.S., Berenguer A., Prats N., Toll A., Hueto J.A. (2017). Targeting metastasis-initiating cells through the fatty acid receptor CD36. Nature.

[54] Lazar I., Clement E., Dauvillier S., Milhas D., Ducoux-Petit M., LeGonidec S., Moro C., Soldan V., Dalle S., Balor S. (2016). Adipocyte Exosomes Promote Melanoma Aggressiveness through Fatty Acid Oxidation: A Novel Mechanism Linking Obesity and Cancer. Cancer Res..

[55] Clement E., Lazar I., Attane C., Carrie L., Dauvillier S., Ducoux-Petit M., Esteve D., Menneteau T., Moutahir M., Le Gonidec S. (2020). Adipocyte extracellular vesicles carry enzymes and fatty acids that stimulate mitochondrial metabolism and remodeling in tumor cells. EMBO J..

[56] Alexandrov L.B., Stratton M.R. (2014). Mutational signatures: The patterns of somatic mutations hidden in cancer genomes. Curr. Opin. Genet. Dev..

[57] Gubin M.M., Zhang X., Schuster H., Caron E., Ward J.P., Noguchi T., Ivanova Y., Hundal J., Arthur C.D., Krebber W.J. (2014). Checkpoint blockade cancer immunotherapy targets tumour-specific mutant antigens. Nature.

[58] Van Allen E.M., Miao D., Schilling B., Shukla S.A., Blank C., Zimmer L., Sucker A., Hillen U., Foppen M.H.G., Goldinger S.M. (2015). Genomic correlates of response to CTLA-4 blockade in metastatic melanoma. Science.

[59] Gros A., Parkhurst M.R., Tran E., Pasetto A., Robbins P.F., Ilyas S., Prickett T.D., Gartner J.J., Crystal J.S., Roberts I.M. (2016). Prospective identification of neoantigen-specific lymphocytes in the peripheral blood of melanoma patients. Nat. Med..

[60] Larkin J., Chiarion-Sileni V., Gonzalez R., Grob J.J., Rutkowski P., Lao C.D., Cowey C.L., Schadendorf D., Wagstaff J., Dummer R. (2019). Five-Year Survival with Combined Nivolumab and Ipilimumab in Advanced Melanoma. N. Engl. J. Med..

[61] Spranger S., Luke J.J., Bao R., Zha Y., Hernandez K.M., Li Y., Gajewski A.P., Andrade J., Gajewski T.F. (2016). Density of immunogenic antigens does not explain the presence or absence of the T-cell-inflamed tumor microenvironment in melanoma. Proc. Natl. Acad. Sci. USA.

[62] Hugo W., Zaretsky J.M., Sun L., Song C., Moreno B.H., Hu-Lieskovan S. (2016). Genomic and Transcriptomic Features of Response to Anti-PD-1 Therapy in Metastatic Melanoma. Cell.

[63] Schreiber R.D., Old L.J., Smyth M.J. (2011). Cancer immunoediting: Integrating immunity’s roles in cancer suppression and promotion. Science.

[64] Riaz N., Havel J.J., Makarov V., Desrichard A., Urba W.J., Sims J.S., Hodi F.S., Martin-Algarra S., Mandal R., Sharfman W.H. (2017). Tumor and Microenvironment Evolution during Immunotherapy with Nivolumab. Cell.

[65] Takeda K., Nakayama M., Hayakawa Y., Kojima Y., Ikeda H., Imai N., Ogasawara K., Okumura K., Thomas D.M., Smyth M.J. (2017). IFN-gamma is required for cytotoxic T cell-dependent cancer genome immunoediting. Nat. Commun..

[66] Mandai M., Hamanishi J., Abiko K., Matsumura N., Baba T., Konishi I. (2016). Dual Faces of IFNgamma in Cancer Progression: A Role of PD-L1 Induction in the Determination of Pro- and Antitumor Immunity. Clin. Cancer Res..

[67] Chang C.C., Pirozzi G., Wen S.H., Chung I.H., Chiu B.L., Errico S., Luongo M., Lombardi M.L., Ferrone S. (2015). Multiple structural and epigenetic defects in the human leukocyte antigen class I antigen presentation pathway in a recurrent metastatic melanoma following immunotherapy. J. Biol. Chem..

[68] Song K.H., Choi C.H., Lee H.J., Oh S.J., Woo S.R., Hong S.O., Noh K.H., Cho H., Chung E.J., Kim J.H. (2017). HDAC1 Upregulation by NANOG Promotes Multidrug Resistance and a Stem-like Phenotype in Immune Edited Tumor Cells. Cancer Res..

[69] Mittal D., Gubin M.M., Schreiber R.D., Smyth M.J. (2014). New insights into cancer immunoediting and its three component phases--elimination, equilibrium and escape. Curr. Opin. Immunol..

[70] Polanczyk M.J., Walker E., Haley D., Guerrouahen B.S., Akporiaye E.T. (2019). Blockade of TGF-beta signaling to enhance the antitumor response is accompanied by dysregulation of the functional activity of CD4(+)CD25(+)Foxp3(+) and CD4(+)CD25(-)Foxp3(+) T cells. J. Transl. Med..

[71] Chockalingam S., Ghosh S.S. (2014). Macrophage colony-stimulating factor and cancer: A review. Tumour Biol..

[72] Reilly S.M., Saltiel A.R. (2017). Adapting to obesity with adipose tissue inflammation. Nat. Rev. Endocrinol..

[73] Lynch L.A., O’Connell J.M., Kwasnik A.K., Cawood T.J., O’Farrelly C., O’Shea D.B. (2009). Are natural killer cells protecting the metabolically healthy obese patient?. Obesity.

[74] James B.R., Tomanek-Chalkley A., Askeland E.J., Kucaba T., Griffith T.S., Norian L.A. (2012). Diet-induced obesity alters dendritic cell function in the presence and absence of tumor growth. J. Immunol..

[75] Tanaka S., Isoda F., Ishihara Y., Kimura M., Yamakawa T. (2001). T lymphopaenia in relation to body mass index and TNF-alpha in human obesity: Adequate weight reduction can be corrective. Clin. Endocrinol..

[76] Yang H., Youm Y.H., Vandanmagsar B., Rood J., Kumar K.G., Butler A.A., Dixit V.D. (2009). Obesity accelerates thymic aging. Blood.

[77] Clements V.K., Long T., Long R., Figley C., Smith D.M.C., Ostrand-Rosenberg S. (2018). Frontline Science: High fat diet and leptin promote tumor progression by inducing myeloid-derived suppressor cells. J. Leukoc. Biol..

[78] Ouchi N., Parker J.L., Lugus J.J., Walsh K. (2011). Adipokines in inflammation and metabolic disease. Nat Rev. Immunol..

[79] Murphy K.A., James B.R., Sjaastad F.V., Kucaba T.A., Kim H., Brincks E.L., Chua S.C., Wilber A., Griffith T.S. (2018). Cutting Edge: Elevated Leptin during Diet-Induced Obesity Reduces the Efficacy of Tumor Immunotherapy. J. Immunol..

[80] Wagner M., Bjerkvig R., Wiig H., Melero-Martin J.M., Lin R.Z., Klagsbrun M., Dudley A.C. (2012). Inflamed tumor-associated adipose tissue is a depot for macrophages that stimulate tumor growth and angiogenesis. Angiogenesis.

[81] Cao Y. (2013). Angiogenesis and vascular functions in modulation of obesity, adipose metabolism, and insulin sensitivity. Cell Metab..

[82] Fox C.J., Hammerman P.S., Thompson C.B. (2005). Fuel feeds function: Energy metabolism and the T-cell response. Nat. Rev. Immunol..

[83] Pearce E.L., Walsh M.C., Cejas P.J., Harms G.M., Shen H., Wang L.S., Jones R.G., Choi Y. (2009). Enhancing CD8 T-cell memory by modulating fatty acid metabolism. Nature.

[84] Bengsch B., Johnson A.L., Kurachi M., Odorizzi P.M., Pauken K.E., Attanasio J., Stelekati E., McLane L.M., Paley M.A., Delgoffe G.M. (2016). Bioenergetic Insufficiencies Due to Metabolic Alterations Regulated by the Inhibitory Receptor PD-1 Are an Early Driver of CD8(+) T Cell Exhaustion. Immunity.

[85] Netea M.G., Quintin J., van der Meer J.W. (2011). Trained immunity: A memory for innate host defense. Cell Host Microbe.

[86] Netea M.G., Dominguez-Andres J., Barreiro L.B., Chavakis T., Divangahi M., Fuchs E., Joosten L.A.B., van der Meer J.W.M., Mhlanga M.M., Mulder W.J.M. (2020). Defining trained immunity and its role in health and disease. Nat. Rev. Immunol..

[87] Christ A., Gunther P., Lauterbach M.A.R., Duewell P., Biswas D., Pelka K., Scholz C.J., Oosting M., Haendler K., Bassler K. (2018). Western Diet Triggers NLRP3-Dependent Innate Immune Reprogramming. Cell.

[88] Zamarron B.F., Mergian T.A., Cho K.W., Martinez-Santibanez G., Luan D., Singer K., DelProposto J.L., Geletka L.M., Muir L.A., Lumeng C.N. (2017). Macrophage Proliferation Sustains Adipose Tissue Inflammation in Formerly Obese Mice. Diabetes.

[89] Stewart. J.H., Levine E.A. (2011). Role of bacillus Calmette-Guerin in the treatment of advanced melanoma. Expert Rev. Anticancer Ther..

[90] Redelman-Sidi G., Glickman M.S., Bochner B.H. (2014). The mechanism of action of BCG therapy for bladder cancer—A current perspective. Nat. Rev. Urol..

[91] Villumsen M., Sorup S., Jess T., Ravn H., Relander T., Baker J.L., Benn C.S., Sorensen T.I., Aaby P., Roth A. (2009). Risk of lymphoma and leukaemia after bacille Calmette-Guerin and smallpox vaccination: A Danish case-cohort study. Vaccine.

[92] Buffen K., Oosting M., Quintin J., Ng A., Kleinnijenhuis J., Kumar V., van de Vosse E., Wijmenga C., van Crevel R., Oosterwijk E. (2014). Autophagy controls BCG-induced trained immunity and the response to intravesical BCG therapy for bladder cancer. PLoS Pathog..

[93] McArthur G.A., Puzanov I., Amaravadi R., Ribas A., Chapman P., Kim K.B., Sosman J.A., Lee R.J., Nolop K., Flaherty K.T. (2012). Marked, homogeneous, and early [18F] fluorodeoxyglucose-positron emission tomography responses to vemurafenib in BRAF-mutant advanced melanoma. J. Clin. Oncol..

[94] Robert C., Grob J.J., Stroyakovskiy D., Karaszewska B., Hauschild A., Levchenko E., Chiarion Sileni V., Schachter J., Garbe C., Bondarenko I. (2019). Five-Year Outcomes with Dabrafenib plus Trametinib in Metastatic Melanoma. N. Engl. J. Med..

[95] Ascierto P.A., McArthur G.A., Dreno B., Atkinson V., Liszkay G., Di Giacomo A.M., Mandala M., Demidov L., Stroyakovskiy D., Thomas L. (2016). Cobimetinib combined with vemurafenib in advanced BRAF(V600)-mutant melanoma (coBRIM): Updated efficacy results from a randomised, double-blind, phase 3 trial. Lancet Oncol..

[96] Ascierto P.A., Dummer R., Gogas H.J., Flaherty K.T., Arance A., Mandala M., Liszkay G., Garbe C., Schadendorf D., Krajsova I. (2020). Update on tolerability and overall survival in COLUMBUS: Landmark analysis of a randomised phase 3 trial of encorafenib plus binimetinib vs vemurafenib or encorafenib in patients with BRAF V600-mutant melanoma. Eur. J. Cancer.

[97] Wellbrock C. (2014). MAPK pathway inhibition in melanoma: Resistance three ways. Biochem. Soc. Trans..

[98] Rambow F., Rogiers A., Marin-Bejar O., Aibar S., Femel J., Dewaele M., Karras P., Brown D., Chang Y.H., Debiec-Rychter M. (2018). Toward Minimal Residual Disease-Directed Therapy in Melanoma. Cell.

[99] Amaral T., Sinnberg T., Meier F., Krepler C., Levesque M., Niessner H., Garbe C. (2017). MAPK pathway in melanoma part II-secondary and adaptive resistance mechanisms to BRAF inhibition. Eur. J. Cancer.

[100] Schneider G., Kirschner M.A., Berkowitz R., Ertel N.H. (1979). Increased estrogen production in obese men. J. Clin. Endocrinol. Metab..

[101] Guillemot-Legris O., Mutemberezi V., Cani P.D., Muccioli G.G. (2016). Obesity is associated with changes in oxysterol metabolism and levels in mice liver, hypothalamus, adipose tissue and plasma. Sci. Rep..

[102] Sottero B., Rossin D., Staurenghi E., Gamba P., Poli G., Testa G. (2019). Omics analysis of oxysterols to better understand their pathophysiological role. Free Radic. Biol. Med..

[103] Pencheva N., Tran H., Buss C., Huh D., Drobnjak M., Busam K., Tavazoie S.F. (2012). Convergent multi-miRNA targeting of ApoE drives LRP1/LRP8-dependent melanoma metastasis and angiogenesis. Cell.

[104] Pencheva N., Buss C.G., Posada J., Merghoub T., Tavazoie S.F. (2014). Broad-spectrum therapeutic suppression of metastatic melanoma through nuclear hormone receptor activation. Cell.

[105] Shao W., Zhu W., Lin J., Luo M., Lin Z., Lu L., Jia H., Qin L., Lu M., Chen J. (2020). Liver X Receptor Agonism Sensitizes a Subset of Hepatocellular Carcinoma to Sorafenib by Dual-Inhibiting MET and EGFR. Neoplasia.

[106] Lin C.Y., Gustafsson J.A. (2015). Targeting liver X receptors in cancer therapeutics. Nat. Rev. Cancer.

[107] Natale C.A., Duperret E.K., Zhang J., Sadeghi R., Dahal A., O’Brien K.T., Cookson R., Winkler J.D., Ridky T.W. (2016). Sex steroids regulate skin pigmentation through nonclassical membrane-bound receptors. eLife.

[108] Cooke P.S., Nanjappa M.K., Ko C., Prins G.S., Hess R.A. (2017). Estrogens in Male Physiology. Physiol. Rev..

[109] Natale C.A., Li J., Zhang J., Dahal A., Dentchev T., Stanger B.Z., Ridky T.W. (2018). Activation of G protein-coupled estrogen receptor signaling inhibits melanoma and improves response to immune checkpoint blockade. eLife.

[110] Singleton K.R., Crawford L., Tsui E., Manchester H.E., Maertens O., Liu X., Liberti M.V., Magpusao A.N., Stein E.M., Tingley J.P. (2017). Melanoma Therapeutic Strategies that Select against Resistance by Exploiting MYC-Driven Evolutionary Convergence. Cell Rep..

[111] Parmenter T.J., Kleinschmidt M., Kinross K.M., Bond S.T., Li J., Kaadige M.R., Rao A., Sheppard K.E., Hugo W., Pupo G.M. (2014). Response of BRAF-mutant melanoma to BRAF inhibition is mediated by a network of transcriptional regulators of glycolysis. Cancer Discov..

[112] Haq R., Shoag J., Andreu-Perez P., Yokoyama S., Edelman H., Rowe G.C., Frederick D.T., Hurley A.D., Nellore A., Kung A.L. (2013). Oncogenic BRAF regulates oxidative metabolism via PGC1alpha and MITF. Cancer Cell.

[113] Gopal Y.N., Rizos H., Chen G., Deng W., Frederick D.T., Cooper Z.A., Scolyer R.A., Pupo G., Komurov K., Sehgal V. (2014). Inhibition of mTORC1/2 overcomes resistance to MAPK pathway inhibitors mediated by PGC1alpha and oxidative phosphorylation in melanoma. Cancer Res..

[114] Zhang G., Frederick D.T., Wu L., Wei Z., Krepler C., Srinivasan S., Chae Y.C., Xu X., Choi H., Dimwamwa E. (2016). Targeting mitochondrial biogenesis to overcome drug resistance to MAPK inhibitors. J. Clin. Investig..

[115] Chi M., Chen J., Ye Y., Tseng H.Y., Lai F., Tay K.H., Jin L., Guo S.T., Jiang C.C., Zhang X.D. (2014). Adipocytes contribute to resistance of human melanoma cells to chemotherapy and targeted therapy. Curr. Med. Chem..

[116] Hopkins B.D., Pauli C., Du X., Wang D.G., Li X., Wu D., Amadiume S.C., Goncalves M.D., Hodakoski C., Lundquist M.R. (2018). Suppression of insulin feedback enhances the efficacy of PI3K inhibitors. Nature.

[117] Shi H., Hugo W., Kong X., Hong A., Koya R.C., Moriceau G., Chodon T., Guo R., Johnson D.B., Dahlman K.B. (2014). Acquired resistance and clonal evolution in melanoma during BRAF inhibitor therapy. Cancer Discov..

[118] Wilmott J.S., Long G.V., Howle J.R., Haydu L.E., Sharma R.N., Thompson J.F., Kefford R.F., Hersey P., Scolyer R.A. (2012). Selective BRAF inhibitors induce marked T-cell infiltration into human metastatic melanoma. Clin. Cancer Res..

[119] Frederick D.T., Piris A., Cogdill A.P., Cooper Z.A., Lezcano C., Ferrone C.R., Mitra D., Boni A., Newton L.P., Liu C. (2013). BRAF inhibition is associated with enhanced melanoma antigen expression and a more favorable tumor microenvironment in patients with metastatic melanoma. Clin. Cancer Res..

[120] Kishton R.J., Sukumar M., Restifo N.P. (2017). Metabolic Regulation of T Cell Longevity and Function in Tumor Immunotherapy. Cell Metab..

[121] Sukumar M., Kishton R.J., Restifo N.P. (2017). Metabolic reprograming of anti-tumor immunity. Curr. Opin. Immunol..

[122] Flaherty S.E., Grijalva A., Xu X., Ables E., Nomani A., Ferrante A.W. (2019). A lipase-independent pathway of lipid release and immune modulation by adipocytes. Science.

[123] Richtig G., Hoeller C., Wolf M., Wolf I., Rainer B.M., Schulter G., Richtig M., Grubler M.R., Gappmayer A., Haidn T. (2018). Body mass index may predict the response to ipilimumab in metastatic melanoma: An observational multi-centre study. PLoS ONE.

[124] Wei S.C., Duffy C.R., Allison J.P. (2018). Fundamental Mechanisms of Immune Checkpoint Blockade Therapy. Cancer Discov..

[125] Wolchok J.D., Rollin L., Larkin J. (2017). Nivolumab and Ipilimumab in Advanced Melanoma. N. Engl. J. Med..

[126] Francisco V., Pino J., Campos-Cabaleiro V., Ruiz-Fernandez C., Mera A., Gonzalez-Gay M.A., Gomez R., Gualillo O. (2018). Obesity, Fat Mass and Immune System: Role for Leptin. Front. Physiol..

[127] Patterson E., Ryan P.M., Cryan J.F., Dinan T.G., Ross R.P., Fitzgerald G.F., Stanton C. (2016). Gut microbiota, obesity and diabetes. Postgrad. Med J..

[128] Zitvogel L., Ma Y., Raoult D., Kroemer G., Gajewski T.F. (2018). The microbiome in cancer immunotherapy: Diagnostic tools and therapeutic strategies. Science.

[129] Gopalakrishnan V., Spencer C.N., Nezi L., Reuben A., Andrews M.C., Karpinets T.V., Prieto P.A., Vicente D., Hoffman K., Wei S.C. (2018). Gut microbiome modulates response to anti-PD-1 immunotherapy in melanoma patients. Science.

[130] Joosse A., Collette S., Suciu S., Nijsten T., Lejeune F., Kleeberg U.R., Coebergh J.W., Eggermont A.M., de Vries E. (2012). Superior outcome of women with stage I/II cutaneous melanoma: Pooled analysis of four European Organisation for Research and Treatment of Cancer phase III trials. J. Clin. Oncol..

[131] Joosse A., Collette S., Suciu S., Nijsten T., Patel P.M., Keilholz U., Eggermont A.M., Coebergh J.W., de Vries E. (2013). Sex is an independent prognostic indicator for survival and relapse/progression-free survival in metastasized stage III to IV melanoma: A pooled analysis of five European organisation for research and treatment of cancer randomized controlled trials. J. Clin. Oncol..

[132] Klein S.L., Flanagan K.L. (2016). Sex differences in immune responses. Nat. Rev. Immunol..

